# Comparison of Face-Touching Behaviors Before and During the Coronavirus Disease 2019 Pandemic

**DOI:** 10.1001/jamanetworkopen.2020.16924

**Published:** 2020-07-29

**Authors:** Yong-Jian Chen, Gang Qin, Jie Chen, Jian-Liang Xu, Ding-Yun Feng, Xiang-Yuan Wu, Xing Li

**Affiliations:** 1 Guangdong Key Laboratory of Liver Disease Research, Department of Medical Oncology, Third Affiliated Hospital of Sun Yat-sen University, Guangzhou, China; 2 Grade 2015, Zhongshan School of Medicine, Sun Yat-sen University, Guangzhou, China; 3 Department of Hepatobiliary Surgery, Third Affiliated Hospital of Sun Yat-sen University, Guangzhou, China; 4 Department of Pulmonary and Critical Care Medicine, Third Affiliated Hospital of Sun Yat-sen University, Guangzhou, China

## Abstract

**Importance:**

There is insufficient evidence on the efficacy of masks in the general population for the prevention of coronavirus disease 2019 (COVID-19) in public areas. Therefore, it is imperative to investigate the association of mandatory mask-wearing policies with behaviors associated with the transmission of COVID-19.

**Objective:**

To assess the association of mask wearing with face-touching behavior among the general population in public areas.

**Design, Setting, and Participants:**

This cross-sectional study used videos recorded in public transportation stations, streets, and parks among the general population in China, Japan, South Korea, Western Europe (ie, England, France, Germany, Spain, and Italy), and the US to analyze mask-wearing and face-touching behavior in public areas. Videos before the COVID-19 pandemic were defined as those recorded from January 2018 to October 2019, and those during the COVID-19 pandemic were defined as those recorded during February 2020 to March 2020 in China, Japan, and South Korea and during March 2020 in Western Europe and the US. Individuals who clearly displayed their face and face-touching behavior were included, and those whose behaviors were influenced by filming or public events were excluded.

**Exposures:**

Mandatory mask-wearing policies enacted at various time points in China, Japan, South Korea, Western Europe, and the US.

**Main Outcomes and Measures:**

Proportion of individuals wearing masks and incidence of face touching.

**Results:**

This study included 4699 individuals before the COVID-19 pandemic and 2887 individuals during the pandemic. During the periods studied, mask wearing increased in all regions except the US, from 20 of 1745 individuals (1.1%) to 1090 of 1097 individuals (99.4%) in mainland China (*P* < .001), 44 of 1422 individuals (3.1%) to 346 of 893 individuals (38.7%) in Japan (*P* < .001), 6 of 717 individuals (0.8%) to 277 of 324 individuals (85.5% ) in South Korea (*P* < .001), 1 of 546 individuals (0.2%) to 6 of 379 individuals (1.6%) in Western Europe (*P* = .02), and 1 of 269 individuals (0.4%) to 4 of 194 individuals (2.1%) in the US (*P* = .17). Surgical masks were predominant in China (989 masks [89.1%]), and fabric masks were predominant in the other regions (Japan: 371 masks [95.1%]; South Korea: 240 masks [84.8%]; Western Europe: 6 masks [85.7%]; US: 5 masks [100%]). Face-touching behaviors decreased from before COVID-19 to during COVID-19 among individuals in China (72 incidences of 1745 observations [4.1%] to 12 incidences of 1097 observations [1.1%]; *P* < .001), South Korea (80 incidences of 717 observations [11.2%] to 7 incidences of 324 observations [2.2%]; *P* < .001), and Europe (62 incidences of 546 observations [11.4%] to 23 incidences of 379 observations [6.1%]; *P* = .01). Logistic regression found that mask wearing was associated with a reduction in face touching in China (odds ratio [OR], 3.91; 95% CI, 2.11-7.24) and South Korea (OR, 6.69; 95% CI, 2.69-16.69) and of touching the nose, mouth, and eyes (China: OR, 8.60; 95% CI, 2.65-27.86; South Korea: OR, 29.27; 95% CI, 1.79-478.22).

**Conclusions and Relevance:**

The findings of this cross-sectional study suggest that mandatory mask-wearing policies were associated with increased mask wearing during the COVID-19 pandemic. Mask wearing was associated with reduced face-touching behavior, especially touching of the eyes, nose, and mouth, which may prevent contact transmission of COVID-19 among the general population in public areas.

## Key Points

**Question:**

Is wearing face masks associated with reduced face-touching behaviors?

**Findings:**

In this cross-sectional study, including 4699 individuals before the coronavirus disease 2019 (COVID-19) pandemic and 2887 individuals during the COVID-19 pandemic, mandatory mask-wearing policies were associated with increased mask wearing among the general population during the COVID-19 pandemic. Wearing masks, either medical or fabric, was associated with reduced face-touching behavior, especially touching of the eyes, nose, and mouth.

**Meaning:**

These findings suggest that mandatory mask-wearing policies were associated with reducing face-touching behavior among the general population in public areas, which may help to prevent contact transmission of COVID-19.

## Teaser

This cross-sectional study examines mask wearing and face-touching behaviors before and during the coronavirus disease 2019 (COVID-19) pandemic in China, Japan, South Korea, Western Europe, and the US.

## Introduction

Coronavirus disease 2019 (COVID-19), which is caused by severe acute respiratory syndrome coronavirus 2 (SARS-CoV-2), has spread worldwide, with more than 10 million cases and 500 000 fatalities as of July 1, 2020.^1^ Droplet transmission is believed to be the dominant route for the spread of COVID-19.^2,3^ Virus-containing droplets (>5 μm) and aerosols (<5 μm) are produced when an infected person coughs or sneezes. Large droplets mainly settle out of the air and cause person or object contamination.^4^ Airborne transmission has been reported in few cases and might occur only with high viral load in confined spaces,^3^ as opposed to public areas, such as streets, parks, and public transport stations. Furthermore, SARS-CoV-2 might be active in droplet contaminated materials for hours to days.^5,6,7^ According to the World Health Organization prevention guideline^8^ and a 2020 meta-analysis,^9^ avoiding touching the eyes, nose, and mouth, maintaining social distancing, and washing hands frequently are the major methods associated with preventing COVID-19 transmission for individuals without respiratory symptoms among the general population. However, the exact contribution of each method is not entirely clear. Face coverings were also recommended to prevent airborne and contact transmission of COVID-19.^2,10^ However, there is currently insufficient evidence for or against the use of masks (medical or other) in healthy individuals in public areas.^11^

An increasing number of governments have enacted mandatory mask-wearing policies for the general population in public areas. However, the mechanisms of the preventive effect associated with masks are poorly understood, which has contributed to limited public acceptance of mandatory mask-wearing policies.^12^ The major mechanisms of the preventive effect associated with masks are considered to be the prevention of direct droplet contamination from the individual to environment.^13,14^ The N95 ventilators for medical use, although efficient in preventing transmission of aerosols,^15,16^ are not necessary or comfortable for the general population. In contrast, fabric masks and surgical masks are considered more cost-effective and comfortable to the general population. Moreover, there is little evidence on the association of masks with reducing contact transmission of SARS-CoV-2 by decreasing face-touching behavior.

In this cross-sectional study, we reviewed individual mask-wearing and face-touching behavior of general populations in China, Japan, South Korea, Western Europe (ie, England, France, Germany, Spain, and Italy), and the US from videos of public areas before and during the COVID-19 pandemic. We analyzed the association of mask wearing with face-touching behavior and discussed the potential associations of different types of masks.

## Methods

This study was approved by the clinical ethics review board of the Third Affiliated Hospital of Sun Yat-sen University and Sun Yat-sen University. Informed consent was waived according to ethical review of biomedical research involving humans by Order of the National Health and Family Planning Commission of the People’s Republic of China No. 11. This study is reported following the Strengthening the Reporting of Observational Studies in Epidemiology (STROBE) reporting guideline.

### Video and Individual Selection

Videos recorded in public areas, including public transportation, streets, and parks, were included and considered as reflecting the actions of the general populations. The videos were for use in tourism marketing or introducing local lifestyles, were made by individuals or mass media, and were searched using Chinese or English from internet video websites, such as Tencent (Tencent Holdings), iQIYI (Baidu), and YouTube (Alphabet). The inclusion criteria were videos recorded in streets, parks, and transport stations that were made with the intention of being used for tourism or lifestyle introduction and that clearly displayed individuals’ faces and face-touching by hands, cellular telephones, or other items, as well as eating. Videos before the COVID-19 pandemic were defined as those recorded from January 2018 to October 2019; videos recorded during the COVID-19 pandemic were defined as those recorded from February 2020 to March 2020 in China, Japan, and South Korea and in March 2020 in Western Europe and the US.

Since the observations of each individual were within 1 minute, the time was insufficient for individuals whose actions were influenced by public events to display the real incidence of face-touching behavior. Public events included speeches, promotions, sports, and other public issues during which people might take pictures or videos, hold hands, or perform another action solely to express their view or response. Thus, the exclusion criteria were using actors; including individuals with obscured faces and hand movements; including individuals who were influenced by film making and public service personnel; involving public events; focusing on people considered as key individuals during the COVID-19 pandemic, such as medical staff, patients, and government officers; and focusing on scenery. Individuals who clearly displayed their faces and face-touching were included.

### Mask Wearing Analysis

Masks were divided into 3 categories: N95, KN95, or KF94 respirators; surgical masks; and fabric masks. Qualified mask wearing was defined as when an individual’s nose and mouth were covered by a mask during the video. Nonqualified mask wearing was defined as exposure of the nose or mouth during the video.

### Face-Touching Behavior Analysis

Face-touching behavior was defined as touching the face with hands, cellular telephones, and other items, as well as eating. Face areas were divided into the forehead, and areas around the eyes, nose, cheek, or mouth. The proportions of mask-wearing individuals were defined as the number of mask-wearing individuals out of the included individuals in each video.

### Statistical Analysis

Differences in characteristics were analyzed using the Pearson χ^2^ or continuity correction for categorical data. Associations of mask wearing with face-touching behavior were modeled using logistic regression models and are reported as odd ratios (ORs) and 95% CIs. Using a forest map, a random-effect model was used to calculate the total effect size of all regions. Excel 2016 spreadsheet software (Microsoft) was used for data input and collation, and R version 3.6.0 (R Project for Statistical Computing) and SPSS version 22.0 (IBM) statistical software were used for descriptive and statistical analysis. *P* values were 2-sided, and *P* < .05 was considered statistically significant.

## Results

### Video Characteristics

We included 14 videos (1551 seconds) from mainland China before the COVID-19 pandemic and 21 videos (2413 seconds) during the pandemic; 7 videos (429 seconds) from Japan before the pandemic and 23 videos (920 seconds) during the pandemic; 2 videos (1200 seconds) from South Korea before the pandemic and 12 videos (299 seconds) during the pandemic; 9 videos (1282 seconds) from Western Europe before the pandemic and 3 videos (472 seconds) during the pandemic; and 6 videos (545 seconds) from the US before the pandemic and 3 videos (357 seconds) during the epidemic (Figure).

**Figure.**
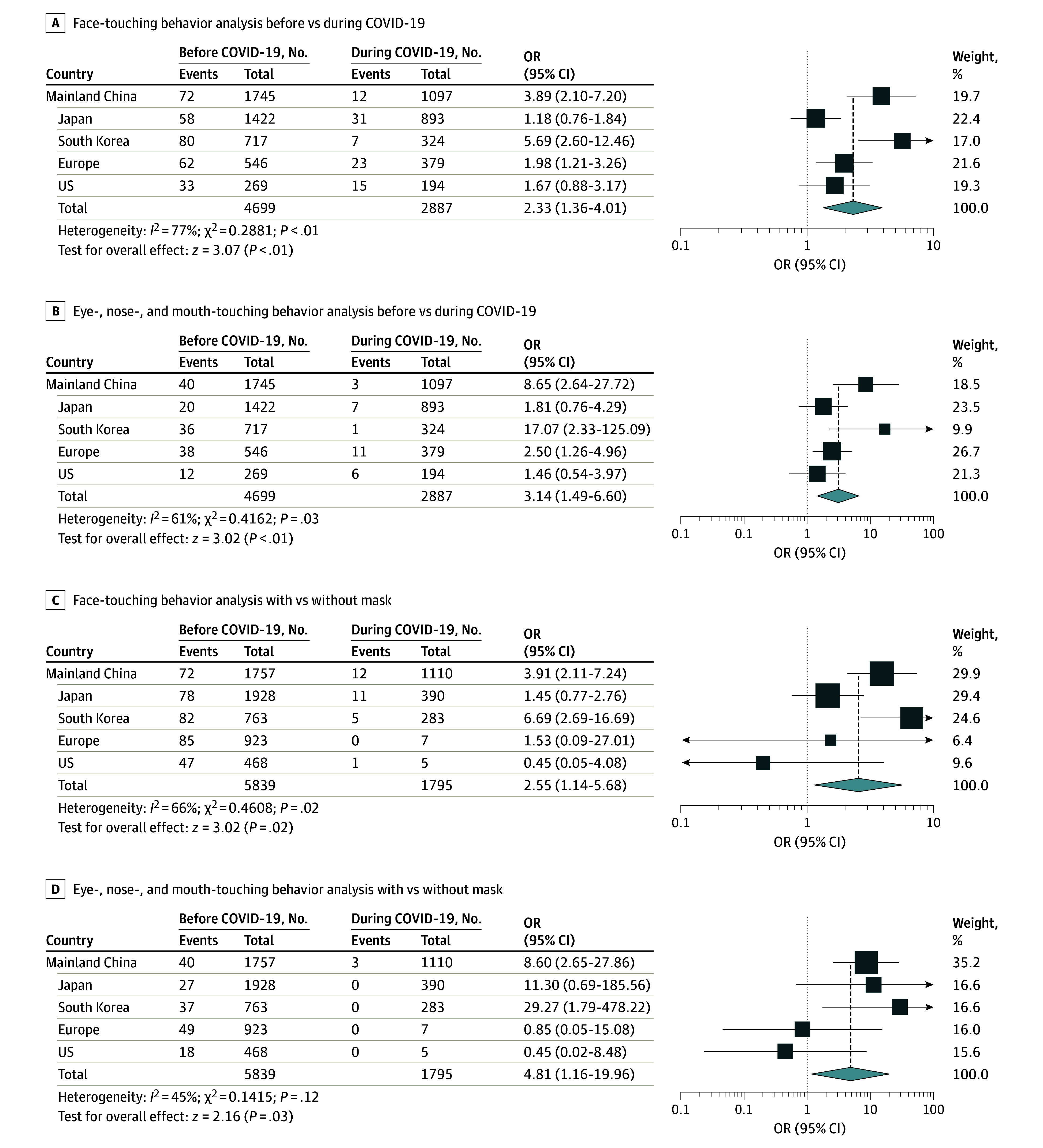
Association of Face-Touching Behaviors and Mask Wearing During the Coronavirus Disease 2019 (COVID-19) Pandemic

This study included 4699 individuals before the COVID-19 pandemic and 2887 individuals during the pandemic. The prepandemic samples included 1745 individuals in China, 1422 individuals in Japan, 717 individuals in South Korea, 546 individuals in Western Europe, and 269 individuals in the US, and the sample for during the COVID-19 pandemic included 1097 individuals in China, 893 individuals in Japan, 324 individuals in South Korea, 379 individuals in Western Europe, and 194 individuals in the US (eFigure in the Supplement).

### Mask-Wearing Rates During the COVID-19 Pandemic

Before the COVID-19 pandemic, the mask-wearing rates were 20 of 1745 individuals (1.1%) in mainland China, 44 of 1422 individuals (3.1%) in Japan, 6 of 717 individuals (0.8%) in South Korea, 1 of 546 individuals (0.2%) in Europe, and 1 of 269 individuals (0.4%) in the US (Table 1), and the qualified mask-wearing rate in mainland China was the lowest among the regions (eTable 1 in the Supplement). During the COVID-19 pandemic, the Chinese government enacted a mandatory mask-wearing policy at the end of January 2020, when the mask-wearing rate increased to 1090 of 1097 individuals (99.4%) (*P* < .001). South Korea enacted a mandatory mask-wearing policy for government employees and also persuaded the general population to wear masks, and the mask-wearing rate in South Korea increased to the second highest, at 277 of 324 individuals (85.5%) (*P* < .001). Furthermore, the Japanese mask-wearing rate increased significantly to 346 of 893 individuals (38.7%) (*P* < .001) (Table 1). However, the mask-wearing rates remained low in Western Europe (6 of 379 individuals [1.6%]; *P* = .02), and there was no statistically significant difference in the US (4 of 194 individuals [2.1%]; *P* = .17). The Chinese general population mainly wore surgical masks (989 masks [89.1%]), while the general populations in other areas mainly wore fabric masks (Japan: 371 masks [95.1%]; South Korea: 240 masks [84.8%]; Western Europe: 6 masks [85.7%]; US: 5 masks [100%]). Goggles were not observed in the included videos (Table 2). The qualified mask-wearing rate increased in mainland China (eTable 1 in the Supplement).

**Table 1.** Mask Wearing Before and During the Coronavirus Disease 2019 Pandemic

Period	Individuals, No. wearing masks/Total No. (%)					*P* value^a^
	Mainland China	Japan	South Korea	Western Europe	US	
Before	20/1745 (1.1)	44/1422 (3.1)	6/717 (0.8)	1/546 (0.2)	1/269 (0.4)	<.001
During	1090/1097 (99.4)	346/893 (38.7)	277/324 (85.5)	6/379 (1.6)	4/194 (2.1)	<.001
*P* value^b^	<.001	<.001	<.001	.02	.17	

^a^ Comparison of mask wearing rates among regions.

^b^ Comparison of mask wearing rates before and during the coronavirus disease 2019 pandemic.

**Table 2.** Mask Type Distribution

Mask types	Masks, No. (%)					*P* value
	Mainland China (n = 1110)	Japan (n = 390)	South Korea (n = 283)	Western Europe (n = 7)	US (n = 5)	
N95, KN95, or KF94 respirator	71 (6.4)	14 (3.6)	38 (13.4)	0	0	<.001
Fabric mask	50 (4.5)	371 (95.1)	240 (84.8)	6 (85.7)	5 (100)	<.001
Surgical mask	989 (89.1)	5 (1.3)	5 (1.7)	1 (14.3)	0	<.001

### Face-Touching Behavior During the COVID-19 Pandemic

Before the COVID-19 pandemic, the incidence of face touching was relatively high in South Korea (80 incidences of 717 observations [11.2%]), Western Europe (62 incidences of 546 observations [11.4%]), and the US (33 incidences of 269 observations [12.3%]) and low in mainland China (72 incidences of 1745 observations [4.1%]) and Japan (58 incidences of 1422 observations [4.1%]). During the COVID-19 pandemic, the incidence of face touching decreased in China (12 incidences of 1097 observations [1.1%]; *P* < .001) South Korea (7 incidences of 324 observations [2.2%]; *P* < .001), and Western Europe (23 incidences of 379 observations [6.1%]; *P* = .01) (Table 3).

**Table 3.** Face-Touching Behavior Incidence Before and During the Coronavirus Disease 2019 Pandemic

Period	Incidents, No./total observations, No. (%)					*P* value^a^
	Mainland China	Japan	South Korea	Western Europe	United States	
Before	72/1745 (4.1)	58/1422 (4.1)	80/717 (11.2)	62/546 (11.4)	33/269 (12.3)	<.001
During	12/1097 (1.1)	31/893 (3.5)	7/324 (2.2)	23/379 (6.1)	15/194 (7.7)	<.001
*P* value^b^	<.001	.48	<.001	.01	.15	

^a^ Comparison of mask wearing rates among regions.

^b^ Comparison of mask wearing rates before and during the coronavirus disease 2019 pandemic.

Based on the difference in mandatory mask-wearing policies among countries, the associations of face-touching reduction and mask-wearing policies were compared among areas. The greatest decreases in incidence of face-touching behaviors were observed in China (OR, 3.89; 95% CI, 2.10-7.20) and South Korea (OR, 5.69; 95% CI, 2.60-12.46). Western Europe had no mandatory mask-wearing policy during March 2020, and face-touching behavior was reduced slightly (OR, 1.98; 95% CI, 1.21-3.26) (Figure, A). With regard to touching the nose, mouth, and eyes, we observed larger decreases in touching behaviors in China (OR, 8.65; 95% CI, 2.64-27.72) and South Korea (OR, 17.07; 95% CI, 2.33-1125.09), although there was also significantly reduced face-touching behavior in Europe (OR, 2.50; 95% CI, 1.26-4.96) (Figure, B).

### Patterns of Face-Touching Behaviors Before and During the COVID-19 Pandemic

Before the COVID-19 pandemic, face touching with hands was common, whereas during the pandemic, face-touching behavior was only reduced in China. Cellular telephone contact on the face relatively increased in China and the US. Other items were mainly food (ie, eating), which showed an increased incidence in China (eTable 2 in the Supplement).

The most frequently touched areas of the face varied among regions. In mainland China, the most frequent area before the COVID-19 pandemic was the nose (22 incidents [30.6%]) and changed to the cheeks (7 incidents [58.3%]) during the pandemic. In Japan, the most frequently touched areas were the forehead and cheek before (forehead: 16 incidences [27.9%]; cheek: 22 incidences [37.9%]) and during the pandemic (forehead: 13 incidences [41.9%]; cheek: 12 incidences [38.7%]). In South Korea, the most frequently touched areas were the mouth (28 incidences [35.0%]) and cheeks (28 incidences [35.0%]) before COVID-19, and changed to the forehead (4 incidences [57.1%]) during the pandemic. Behaviors did not change in the US. Mouth touching (including eating) decreased significantly only in Europe during the pandemic (before: 29 incidences [46.7%]; during: 4 incidences [17.4%]), where the incidence of mouth touching was the highest among all the regions (Table 4).

**Table 4.** Face Areas Being Touched Before and During the Coronavirus Disease 2019 Pandemic

Region	No. (%)					*P* value^a^
	Eye	Nose	Forehead	Mouth	Cheek	
**Mainland China**						
Before (n = 72)	6 (8.3)	22 (30.6)	18 (25.0)	12 (16.7)	16 (22.2)	.01
During (n = 12)	0	0	2 (16.7)	3 (25.0)	7 (58.3)	.08
*P* value^b^	.59	.03	.72	.45	.02	
**Japan**						
Before (n = 58)	1 (1.7)	10 (17.2)	16 (27.6)	9 (15.5)	22 (37.9)	<.001
During (n = 31)	0	4 (12.9)	13 (41.9)	3 (9.7)	12 (38.7)	<.001
*P* value^b^	.99	.76	.24	.53	.99	
**South Korea**						
Before (n = 80)	2 (2.5)	6 (7.5)	16 (20.0)	28 (35.0)	28 (35.0)	<.001
During (n = 7)	0	1 (14.3)	4 (57.1)	0	2 (28.6)	.04
*P* value^b^	.99	.46	.046	.09	.99	
**Western Europe**						
Before (n = 62)	2 (3.2)	7 (11.3)	11 (17.7)	29 (46.7)	14 (22.6)	<.001
During (n = 23)	2 (8.7)	5 (21.7)	5 (21.7)	4 (17.4)	7 (30.4)	.47
*P* value^b^	.30	.29	.76	.02	.39	
**US**						
Before (n = 33)	0	1 (3.0)	10 (30.3)	11 (33.3)	11 (33.3)	<.001
During (n = 15)	0	2 (13.3)	1 (6.7)	4 (26.7)	6 (40.0)	.03
*P* value^b^	NA	.23	.14	.75	.75	

^a^ Comparison of touching rates among face regions.

^b^ Comparison of indicated face area touching rates before and during the coronavirus disease 2019 pandemic.

### Association of Mask Wearing With Decreased Face-Touching Behaviors

General populations wearing masks displayed significant reductions in face-touching behaviors, with the exception of eye-touching behaviors (eTable 3 in the Supplement). Among the included populations, face-touching behaviors were significantly reduced in China (OR, 3.91; 95% CI, 2.11-7.24) and South Korea (OR, 6.69; 95% CI, 2.69-16.69) (Figure, C). Moreover, China and South Korea had more significant reductions in touching of the nose, mouth, and eyes (China: OR, 8.60; 95% CI, 2.65-27.86; South Korea: OR, 29.27; 95% CI, 1.79-478.22). The results of the European and US populations were not significant, possibly owing to the limited sample sizes in the mask-wearing cohorts (Figure, D). Thus, mask wearing was associated with a reduction in face-touching behaviors, especially touching of the nose, mouth, and eyes.

## Discussion

Masks have been reported to be useful in preventing influenza-like illnesses^17,18^ and coronavirus,^9,19^ although the mechanisms are largely unknown. In public areas, individuals infected with SARS-CoV-2, with or without symptoms, might contaminate their environment, which would subsequently contaminate the hands of the general population.^3,20^ This type of transmission might be more important in open areas, such as streets, parks, and public transport stations. Contamination of the face via hands and items might be a critical transmission route of SARS-CoV-2 in the general population in public areas.^8^ Therefore, decreasing facial contamination is considered to be effective in preventing COVID-19 in the general population. This cross-sectional study found that face-touching behaviors were reduced during the COVID-19 pandemic, especially among people in mainland China and South Korea, which were the first areas to introduce mandatory mask-wearing policies that were associated mitigating the risk of COVID-19 within 2 months.^1^ With the spread of COVID-19, mandatory mask-wearing policies for the general population have gained increasing interest, in particular from governments in Western Europe, where the COVID-19 pandemic was mitigated within approximately 3 months.^1^ Thus, the reduction of face-touching behaviors by mask wearing could contribute to curbing the COVID-19 pandemic.

It is possible that masks could themselves become contaminated. In our study, masks were seen to be touched by hands or other items. Furthermore, clothing, masks, goggles, hats, uncovered skin, and hair could be contaminated by environmental SARS-CoV-2 through touching.^5,6,7^ Disposable personal protective equipment has been used to protect medical stuff^15^; however, this is not considered to be cost-effective or necessary for the general population. Instead, for the general population, we recommend households set aside semicontaminated zone where they can dispose of used masks, sterilize goggles and skin, wash clothes and fabric masks, and take a shower before entering their home, the clean zone.

The findings of this study suggest that fabric masks were associated with reduced face-touching behaviors to a similar degree as surgical masks. In mainland China, most of the masks were surgical masks, while fabric masks were mostly used in South Korea; these differences may be due to cultural and economic reasons. However, their associations with reducing face-touching behaviors were similar. Although fabric masks do not present the same protective barrier against SARS-CoV-2 droplets as surgical masks and N95 ventilators,^14,15,16^ they are considered to play a fundamental role for use by the general population in public areas. As a result, the World Health Organization has given recommendations for safely wearing fabric masks to efficiently protect the general population.^8^

### Limitations

This study has several limitations. Enclosed spaces, such as offices, restaurants, museums, and schools, were not included in our study. Thus, a reduction in face-touching behaviors by mask wearing was not determined in enclosed spaces. Moreover, this study included relatively fewer videos from South Korea, Western Europe, and the US during the COVID-19 pandemic owing to a critical decrease in videos meeting the inclusion criteria. Individuals with masks in Western Europe and the US were too few to achieve reliable results on the association of mask-wearing and face-touching behaviors in those areas. Furthermore, our study did not directly prove the mitigating role of masks in the COVID-19 pandemic. Prospective cohort studies are needed to examine the associations of different types of masks with the prevention of COVID-19.^9^

## Conclusions

This cross-sectional study found that mandatory mask-wearing policies increased the mask-wearing rate among the general population during the COVID-19 pandemic. Wearing either a medical or fabric mask was associated with reduced face-touching behaviors, which might prevent transmission of COVID-19 among the general population in public areas.

## Notes

Supplement. **eFigure.** Video and Individual Selection Flowchart Before and During the Coronavirus Disease 2019 (COVID-19) Pandemic **eTable 1.** Mask Wearing Qualification Rate Before and During the Coronavirus Disease 2019 Pandemic **eTable 2.** Patterns of Face Touching Behavior Before and During the Coronavirus Disease 2019 pandemic **eTable 3.** Face Areas Being Touched With or Without Mask
