# Supplementary material for: Comparison of Face-Touching Behaviors Before and During the Coronavirus Disease 2019 Pandemic
Source: JAMA Netw Open. 2020 Jul 29;3(7):e2016924. doi: 10.1001/jamanetworkopen.2020.16924 (PMC12124488; doi:10.1001/jamanetworkopen.2020.16924)
Supplement: Supplement. — eFigure. Video and Individual Selection Flowchart Before and During the Coronavirus Disease 2019 (COVID-19) Pandemic eTable 1. Mask Wearing Qualification Rate Before and During the Coronavirus Disease 2019 Pandemic eTable 2. Patterns of Face Touching Behavior Before and During the Coronavirus Disease 2019 pandemic eTable 3. Face Areas Being Touched With or Without Mask [file jamanetwopen-e2016924-s001.pdf]

Supplementary Online Content

Chen Y-J, Qin G, Chen J, et al. Comparison of face-touching behaviors before and during the coronavirus disease 2019 pandemic. *JAMA Netw Open*. 2020;3(7):e2016924. doi:10.1001/jamanetworkopen.2020.16924

eFigure. Video and Individual Selection Flowchart Before and During the Coronavirus Disease 2019 (COVID-19) Pandemic

eTable 1. Mask Wearing Qualification Rate Before and During the Coronavirus Disease 2019 Pandemic

eTable 2. Patterns of Face Touching Behavior Before and During the Coronavirus Disease 2019 pandemic

eTable 3. Face Areas Being Touched With or Without Mask

This supplementary material has been provided by the authors to give readers additional information about their work.

© 2020 Chen Y-J et al. *JAMA Network Open.*

eFigure. Video and Individual Selection Flowchart Before and During the Coronavirus Disease 2019 (COVID-19) Pandemic

A. Video and individual selection flowchart before COVID-19

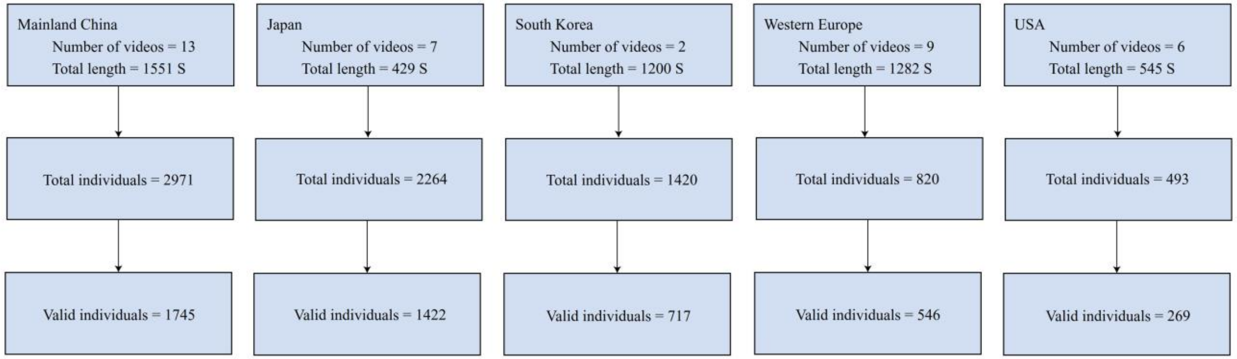

B. Video and individual selection flowchart after COVID-19

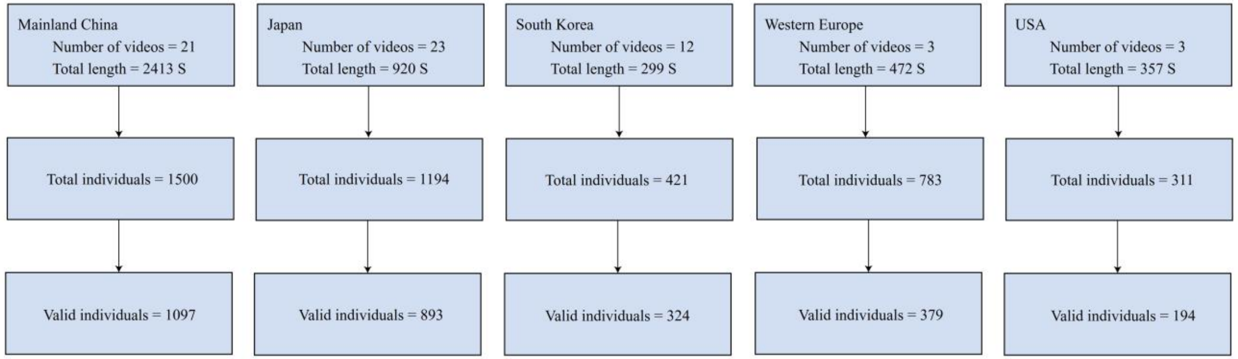

© 2020 Chen Y-J et al. *JAMA Network Open.*

eTable 1. Mask Wearing Qualification Rate Before and During the Coronavirus Disease 2019 Pandemic

	Incidents, No./total observations, No. (%)					
Period	Mainland China	Japan	South Korea	Western Europe	USA	*P* value[a]
Before	17/20 (85.0)	42/44 (95.5)	6/6 (100.0)	1/1 (100.0)	1/1 (100.0)	0.558
During	1068/1090 (98.0)	331/346 (95.7)	269/277 (97.1)	6/6 (100.0)	4/4 (100.0)	0.207
P value[b]	0.669	0.992	0.960	0.999	0.999	

[a]Comparison of mask wearing qualification rates among regions; [b]Comparison of mask wearing rates before and during the coronavirus disease 2019 pandemic

© 2020 Chen Y-J et al. *JAMA Network Open.*

eTable 2. Patterns of Face Touching Behavior Before and During the Coronavirus Disease 2019 pandemic

Region	No. (%)			*P* value[a]
	Hands	Cellphone	Others	
Mainland China				
Before (n = 72)	60 (83.3)	10 (13.9)	2 (2.8)	<0.001
During (n = 12)	2 (16.7)	7 (58.3)	3 (25.0)	0.072
P value[b]	<0.001	0.002	0.020	
Japan				
Before (n = 58)	44 (75.9)	11 (19.0)	3 (5.2)	<0.001
During (n = 31)	21 (67.7)	9 (29.0)	1 (3.2)	<0.001
P value[b]	0.458	0.297	0.999***	
Southern Korea				
Before (n = 80)	46 (57.5)	12 (15.0)	22 (27.5)	<0.001
During (n = 7)	5 (71.4)	2 (28.5)	0 (0.0)	0.017
P value[b]	0.695	0.313	0.184	
USA				
Before (n = 33)	23 (69.7)	1 (3.0)	9 (27.3)	<0.001
During (n = 15)	7 (46.7)	5 (33.3)	2 (13.3)	0.139
P value[b]	0.198	0.008	0.462	
Europe				
Before (n = 62)	30 (48.4)	10 (16.1)	22 (35.5)	0.001
During (n = 23)	17 (73.9)	2 (8.7)	3 (13.0)	<0.001
P value[b]	0.049	0.499	0.060	

[a]Comparison of face touching patterns; [b]Comparison of indicated face touching pattern before and during the coronavirus disease 2019 pandemic; *** > 0.9999.

© 2020 Chen Y-J et al. *JAMA Network Open.*

eTable 3. Face Areas Being Touched With or Without Mask

Mask wearing	No. (%)					
	Eye	Nose	Forehead	Mouth	Cheek	*P* value[a]
Mask (n = 1795)	0 (0.0)	0 (0.0)	9 (0.005)	3 (0.002)	17 (0.009)	< 0.001
No mask (n = 5839)	13 (0.002)	58 (0.010)	87 (0.015)	100 (0.017)	108 (0.018)	< 0.001
P value[b]	0.099	< 0.001	0.001	< 0.001	0.008	

[a]Comparison of touching rates among face regions; [b]Comparison of indicated face area touching rates with or without mask.

© 2020 Chen Y-J et al. *JAMA Network Open.*
